# P02-027 - Quality of life in CAPS treated by Canakinumab

**DOI:** 10.1186/1546-0096-11-S1-A134

**Published:** 2013-11-08

**Authors:** C Marsaud, I Marie, I Koné-Paut

**Affiliations:** 1 Pediatrie générale, Bicêtre hospital, France; 2 CEREMAI, Le kremlin-Bicêtre, Bicêtre hospital, France

## Introduction

The impact of cryopyrin associated periodic syndrome (CAPS) on quality of life (QoL) is very high. Constitutional symptoms, extreme fatigue, chronic pain and physical limitation lead to severe restriction of daily activities and social life. In addition neurological and sensory impairments may aggravate social exclusion of these patients.

## Objectives

To evaluate the quality of life, the social and professional impact of a small cohort of CAPS patients after a long-term treatment with Canakinumab, a selective anti interleukine1β (IL-1β) monoclonal antibody.

## Methods

Patients were those first included in the pivotal (D2304) and in the extension (D2306) Canakinumab/CAPS study [1] and followed in the reference center for autoinflammatory diseases at Bicêtre Hospital (Paris). All carried a heterozygous mutation in the NLPR3 gene and had received Canakinumab (dose of 150 mg every 8 weeks or 2mg/kg for patient weight under 40kg). All patients had been followed prospectively and their quality of life had been evaluated regularly with generic questionnaires: Functional Assessment of Chronic Illness Therapy-fatigue (FACIT-F), 36-item Short Form health survey (SF-36) and Health Assessment Questionnaire (HAQ) for adults. Furthermore supplementary questions regarding their social activities and how they deal with their treatment were added.

## Results

7 patients were analyzed (3males/4females, age 24 to 63-years-old). The mean time of follow-up from initial treatment to last visit was 4.8 +/-0.8 years. All patients were in remission defined as a physician global assessment of disease activity that was minimal or absent, with skin rash that was minimal or absent, and serum values of CRP and/or SAA in the normal range. A significant and sustained improvement in physical SF-36 (37.7 from baseline vs 49,7 at end of D2306 study and 48.6 points at last visit, p<0.05) and FACIT-F (25.1 vs 42,6 at end of D2306 study and 42.3 points at last visit p<0.05) scores was found, with scores approaching scores in American general population. They notified modifications in their social lives: 1 patient restarted professional activity, 4 patients restarted with sportive activity and 1 patient stopped smoking. Concerning their affective lives, 1 single man patient lives actually in a relationship, and 1 patient divorced. No local reaction after injection was observed during the follow-up. Only 2 patients realized subcutaneous injections by their own, the 5 others needed a nurse for the injection. Doses or intervals of injections were modified in 4 patients because of incomplete remission during follow-up (1 received 300 mg instead 150mg, and 3 received injections at 6 or 7 interval weeks).

## Conclusion

Long-term treatment with Canakinumab in patients with CAPS allows a sustained and significant improvement of quality of life with the generic questionnaire FACIT-F and SF-36. Patients show important modification in their social and professional lives.

## Disclosure of interest

None declared.
